# An update on mechanisms of pruritus and their potential treatment in primary cutaneous T-cell lymphoma

**DOI:** 10.1007/s10238-023-01141-x

**Published:** 2023-08-09

**Authors:** Man Hu, Jörg Scheffel, Daniel Elieh-Ali-Komi, Marcus Maurer, Tomasz Hawro, Martin Metz

**Affiliations:** 1 Institute of Allergology, Charité – Universitätsmedizin Berlin, Corporate Member of Freie Universität Berlin, Humboldt-Universität Zu Berlin, Hindenburgdamm 27, 12203 Berlin, Germany; 2 Fraunhofer Institute for Translational Medicine and Pharmacology ITMP, Allergology and Immunology, Berlin, Germany; 3 Department of Dermatology, Allergology and Venereology, Institute and Comprehensive Center for Inflammation Medicine, University Medical Center Schleswig-Holstein, Lübeck, Germany

**Keywords:** Cutaneous T cell lymphoma, Itch, Mycosis fungoides, Pruritus, Sézary syndrome

## Abstract

Primary cutaneous T-cell lymphomas (CTCL), which include mycosis fungoides (MF) and Sézary syndrome (SS), are a group of lymphoproliferative disorders characterized by clonal accumulation of neoplastic T-lymphocytes in the skin. Severe pruritus, one of the most common and distressing symptoms in primary CTCL, can significantly impair emotional well-being, physical functioning, and interpersonal relationships, thus greatly reducing quality of life. Unfortunately, effectively managing pruritus remains challenging in CTCL patients as the underlying mechanisms are, as of yet, not fully understood. Previous studies investigating the mechanisms of itch in CTCL have identified several mediators and their corresponding antagonists used for treatment. However, a comprehensive overview of the mediators and receptors contributing to pruritus in primary CTCL is lacking in the current literature. Here, we summarize and review the mediators and receptors that may contribute to pruritus in primary CTCL to explore the mechanisms of CTCL pruritus and identify effective therapeutic targets using the PubMed and Web of Science databases. Studies were included if they described itch mediators and receptors in MF and SS. Overall, the available data suggest that proteases (mainly tryptase), and neuropeptides (particularly Substance P) may be of greatest interest. At the receptor level, cytokine receptors, MRGPRs, and TRP channels are most likely important. Future drug development efforts should concentrate on targeting these mediators and receptors for the treatment of CTCL pruritus.

## Introduction

Per definition, primary cutaneous lymphomas are non-Hodgkin lymphomas in the skin without evidence of extracutaneous disease at the time of diagnosis [1]. The group of cutaneous lymphomas consists of primary cutaneous T cell lymphoma (CTCL) and primary cutaneous B cell lymphoma (CBCL) subtypes, with CTCL accounting for about 75–80% of all cutaneous lymphomas worldwide [1]. Among all CTCL, mycosis fungoides (MF) is the most common variant, representing approximately 60% of all cases [1]. Variants of MF include folliculotropic MF, pagetoid reticulosis, and granulomatous slack skin [2, 3]. The other classic type of CTCL, Sézary syndrome (SS), accounts for less than 3% of all CTCL [1], and is a rare, aggressive leukemic subtype of CTCL of slow onset [4, 5]. CBCL, constituting ∼20% to 25% of all primary cutaneous lymphomas, is subdivided into three main subtypes, marginal zone B-cell lymphoma, follicle center lymphoma and diffuse large B-cell lymphoma [6]. Even though about 40% of CBCL patients report localized pruritus [7], the clinical significance of itch seems to be of less importance in CBCL patients than in CTCL patients.

Pruritus is among the most severe and challenging clinical symptoms in CTCL patients [8–11]. It affects up to 88% of all CTCL patients, 61% of MF patients, and 94% of patients with SS [9, 12]. The average pruritus intensity, as assessed by a visual analogue scale ranging from 0 (no itch) to 10 (unbearable itch) is reported to increase in MF with progression of the disease from 3.4 in early stage disease (Ia-IIa) to 6.6 in late stage (IIb-IVb), and 7.7 in SS patients [9]. Pruritus in CTCL is usually long lasting and refractory to standard treatment with topical steroids or oral antihistamines [6, 13–15]. Overall, it has been shown that pruritus is one of the main factors affecting the health-related quality of life and mental health of patients with CTCL [16–18].

While the mechanisms underlying pruritus in CTCL are still poorly understood, the increasing information on pruritus-associated mediators and receptors allows to speculate on their possible roles on pruritus in CTCL. In this review, we aim to summarize published evidence on mediators and receptors that are potentially involved in CTCL-associated pruritus and could serve as antipruritic targets.

## Methods

**Fig. 1:**
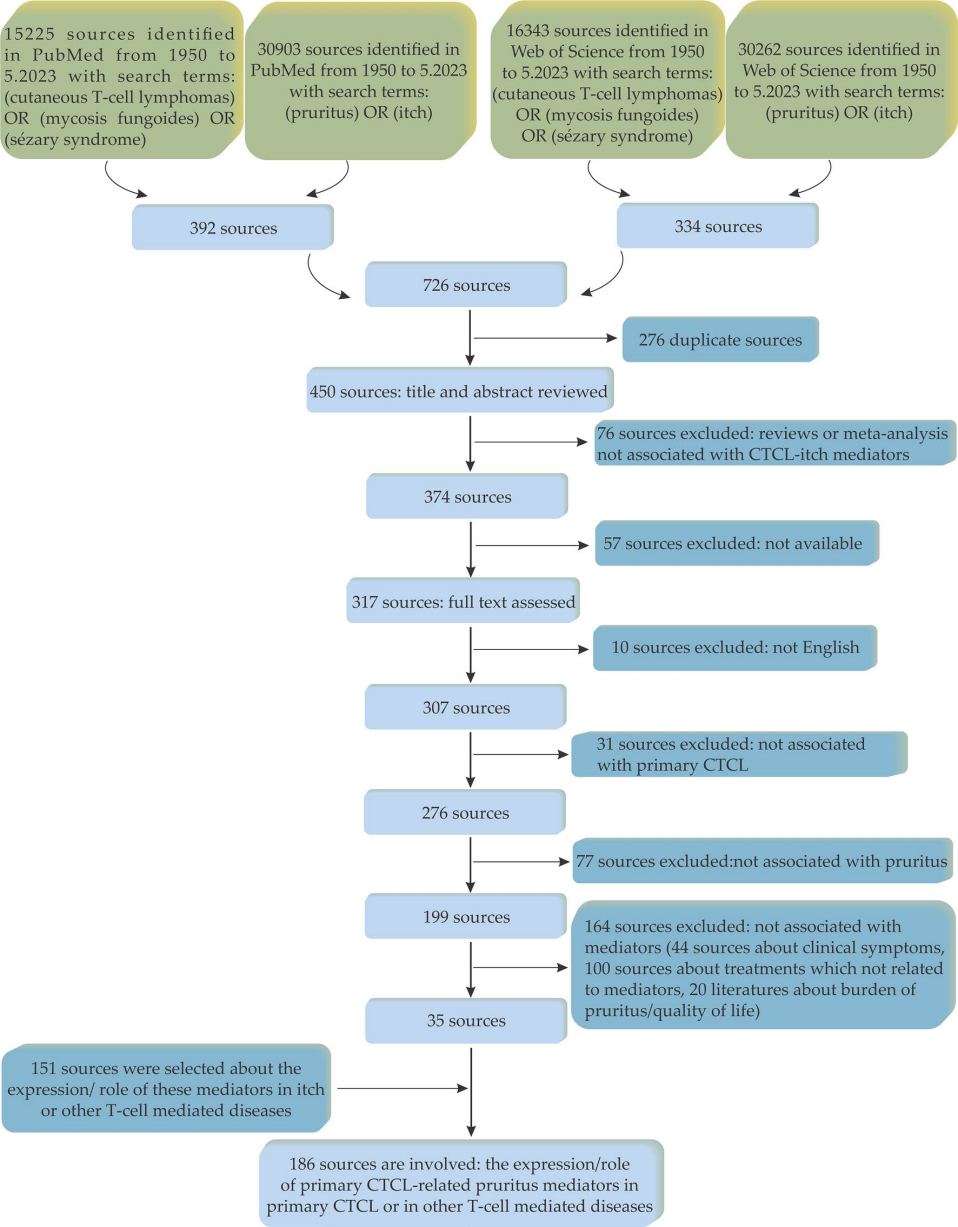
Flow diagram representing the inclusion and exclusion criteria considered to select the relevant references. Abbreviations: CTCL, cutaneous T-cell lymphomas

PubMed and Web of Science were searched using the terms ‘itch’, ‘pruritus’, ‘cutaneous T-cell lymphomas’, ‘Mycosis Fungoides’ and ‘Sézary syndrome’. All relevant published papers available from 1950 to May 2023 were included. Figure 1 represents the flowchart of inclusion and exclusion criteria considered to select the relevant references.

## Cytokines and chemokines

### Interleukin-4 and interleukin-13

**Fig. 2:**
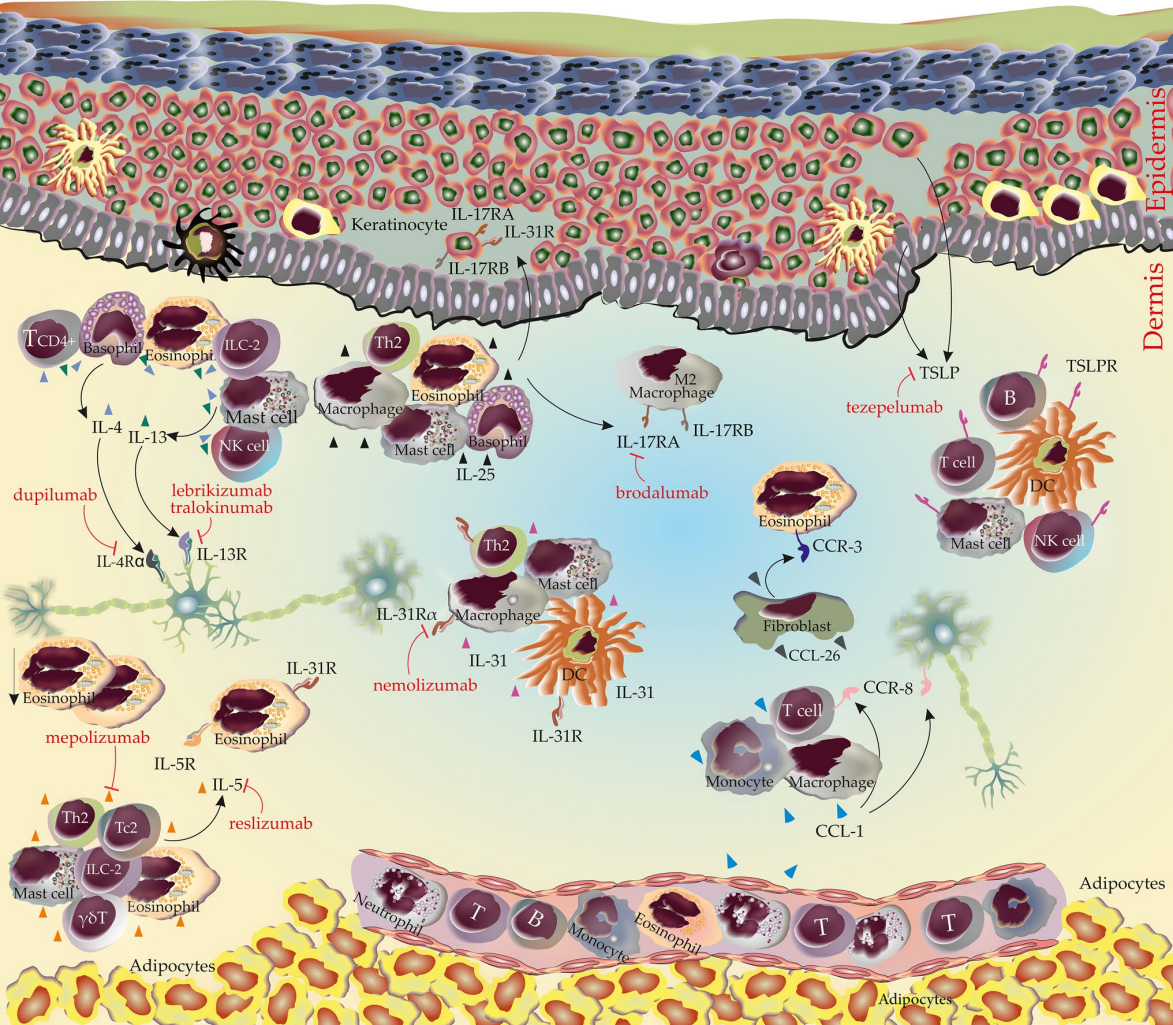
Cytokines, chemokines, and their receptors potentially involved in CTCL pruritus. mAbs as treatment are shown in red with blocking symbol. All cells releasing the cytokines or express the receptors are shown for each subsection. Abbreviations: CCL, Chemokine C–C motif ligand; CCR, CC chemokine receptor; IL, Interleukin; ILC-2, Group 2 innate lymphoid cells; TSLP, Thymic stromal lymphopoietin

Interleukin (IL)-4 and IL-13 are cytokines that have overlapping secondary structural features and share 25% sequence homology [19, 20]. They can be produced and released by various cells, including CD4 + T cells, basophils, eosinophils, mast cells, natural killer T cells, and group 2 innate lymphoid cells [21, 22]. IL-4 signals via type I or type II receptors, consisting either of IL-4Rα paired with common γ-chain (type I; IL-4Rα/ γc) or IL-4Rα paired with IL-13Rα1 (type II; IL-4Rα/IL-13Rα1). While the IL-4Rα/ γc receptor complex only binds IL-4, IL-4Rα/IL-13Rα1 can also interact with IL-13, which also binds and signals through IL-13Rα2 [22–24] (Fig. 2).

It has been shown that transgenic mice overexpressing IL-4 in the epidermis spontaneously develop a pruritic inflammatory skin disease [25]. Importantly, IL-4 as well as IL-13 have also been found to directly activate a subset of sensory neurons, thereby sensitizing them for subsequent stimulation with pruritogenic mediators such as IL-31, histamine, thymic stromal lymphopoietin (TSLP) or chloroquine [26]. Consistent with these findings, clinical trials in patients with moderate to severe atopic dermatitis (AD) have shown that the monoclonal antibody to IL-4Rα, dupilumab, effectively reduces pruritus [27–29], and the anti-IL-13 antibodies lebrikizumab and tralokinumab lead to an improvement of pruritus in moderate to severe AD [30–32] (Table 1). In chronic prurigo and chronic pruritus of unknown origin, dupilumab has also been proven to be efficacious in a large number of case reports and case series [33]. Furthermore, patients suffering from chronic pruritus of unknown origin or AD benefit from inhibition of JAK1, which is the major signaling component in type I and type II IL-4R signaling [26]. All together, these findings suggest that IL-4 and IL-13 can contribute to and promote chronic pruritus.

**Table 1:** Potential drivers of itch and therapeutic targets for the treatment of pruritus in CTCL

Mediator	Drug	Effects on pruritus	Effect on pruritus in CTCL
Cytokines and chemokines			
IL-4	Dupilumab	Significant relief in AD [27–29]	Significant relief [37, 38], No improvement [40–43, 47, 49]
IL-13	Lebrikizumab	Significant relief in AD [28, 30]	Unknown
IL-13	Tralokinumab	Significant relief in AD [28, 31, 32]	Unknown
IL-5	Reslizumab	Significant relief in hypereosinophilic syndrome [62]	Unknown
IL-5	Mepolizumab	Significant relief in hypereosinophilic syndrome [63] and Wells syndrome [64]	Unknown
IL-25	None available	Unknown	Unknown
IL-31	Nemolizumab	Significant relief in AD [90–92] and prurigo nodularis [93, 94]	Unknown
CCL-1	None available	Unknown	Unknown
CCL-26	None available	Unknown	Unknown
TSLP	Tezepelumab	Minor improvement in AD [119]	Unknown
Neuropeptides and neurotrophins			
NGF	CT327	Significant relief in psoriasis [132]	Unknown
SP	Aprepitant	Significant relief in PN-associated itch [145], brachioradial pruritus [146, 152], drugs [147–149], paraneoplastic pruritus [150], psoriasis [151], solid tumors [152], systemic diseases [153] such as chronic kidney disease, hyperuricemia, iron deficiency. No improvement in PN [154] and AD [155]	Significant relief [165–170], No improvement [171]
SP	Serlopitant	Significant relief in PN [156] psoriasis [157, 158], CPUO [160]. No improvement in epidermolysis bullosa [146]	Unknown
SP	Tradipitant	Significant relief in AD [161]	Unknown
SP	Orvepitant	Significant relief in EGFRi-induced intense pruritus [163]	Unknown
VEGF	Bevacizumab	Significant relief in chronic pruritus [179]	Unknown
Proteases			
KLK5	None available	Unknown	Unknown
Tryptase	MTPS9579A	Unknown (ongoing phase 2 trial in CSU, NCT05129423)	Unknown
Itch associated receptors and ion channels			
MRGPRs	None available	Unknown	Unknown
Opioid	Naltrexone	Significant relief in uremia [216], psoriasis [216, 221], PN [216], cholestatic itch [216, 219] and lichen simplex chronicus [221]	Significant relief [216, 248–250], No improvement [251]
Opioid	Nalmefene	Significant relief in AD [217, 218], chronic urticaria [217, 218]	Unknown
Opioid	Morphine	Elicits pruritus [226, 227]	Unknown
Opioid	Difelikefalin	Significant relief in chronic kidney disease [228–230]	Unknown
Opioid	Nalfurafine	Significant relief in hemodialysis patients [231, 233–235] and chronic liver disease [232, 235, 236]	Unknown
Opioid	Nalbuphine	Significant relief in morphine-induced pruritus [237, 238], PN [239] and uremia [240, 241]	Unknown
Opioid	Butorphanol	Significant relief in morphine-related pruritus [242, 243], cholestatic pruritus [244], postherpetic itch [245], PN [246], systemic diseases-related pruritus [246, 247]	Unknown
Opioid	Naloxone	Significant relief in cholestatic pruritus [222–224]	Significant relief [251]
PAR-2	None available	Unknown	Unknown
TRP channels	PAC-14028	Significant relief in AD [276, 277]	Unknown

AD, atopic dermatitis; CCL, chemokine C–C motif ligand; CPUO, chronic pruritus of unknown origin; CTCL, cutaneous T-cell lymphomas; EGFRi, epidermal growth factor receptor inhibitors; IL, Interleukin; KLK5, Kallikrein-related peptide 5; MRGPRs, mas-related G protein-coupled receptors; NGF, nerve growth factor; PAR-2, protease-activated receptor 2; PN, prurigo nodularis; SP, substance P; TRP Channels, transient receptor potential channels; TSLP, thymic stromal lymphopoietin

In CTCL, studies have shown that IL-4 may be an early indicator of disease progression. The levels of IL-4 in peripheral blood mononuclear cells (PBMC) of patients with SS and erythrodermic MF were significantly higher than those in control groups [34, 35]. The expression level of IL-13 mRNA in the lymph nodes of SS patients was significantly higher than that in other lymphomas, including diffuse large cell lymphomas, follicular lymphomas, peripheral T-cell lymphomas, anaplastic large cell lymphomas, and tumor-free reactive lymph nodes [36]. Recent reports on patients with CTCL indicate that dupilumab treatment can improve pruritus in CTCL [37, 38]. It has to be noted, however, that several studies reported about the development or exacerbation of CTCL after dupilumab treatment [39–49]. One suggested mechanism for the potential acceleration of CTCL progression by dupilumab involves an increase in the availability of IL-13 for binding at the IL-13 receptor (IL-13R) α2 site due to the indirect blockade of the IL-13Rα1 site by dupilumab [42]. CTCL cells have been observed to produce higher levels of IL-13 and IL-13Rα2 compared to normal skin, resulting in self-sustaining growth signals for tumors [42]. The blocking of the α subunit of the IL-4R by dupilumab effectively enhances the pool of available IL-13, which can then contribute to the promotion of tumorigenic pathways [42, 50]. Another hypothesis suggests that the worsening of CTCL might be linked to the direct advancement of malignant T-cell clones, which correlates with the depletion of tumor-suppressive, tumor-infiltrating lymphocytes [41]. Moreover, tumor cells may develop resistance to the effects of dupilumab, leading to the emergence of a clone that is no longer responsive to treatment [41]. Therefore, in CTCL patients, the potential symptomatic benefit of dupilumab must be weighed against the risk of disease progression [50, 51].

### Interleukin-5

Interleukin-5 belongs to the common β chain (βc) signaling cytokine family including IL-3 and GM-CSF, which share the βc for signaling, while the IL-5R specifically interacts with IL-5 [52–54]. The major cellular sources of IL-5 are Th2 cells, Tc2 cells, mast cells, eosinophils, and γδT cells [55]. In addition, group 2 innate lymphoid cells can produce high levels of IL-5 when properly stimulated [56]. While the IL-5R subunit is strongly expressed by eosinophils and basophils, mast cells exhibit a rather low expression [52, 57]. (Fig. 2).

IL-5 plays a key role in the production and function of eosinophils. Monoclonal antibodies against IL-5 (mepolizumab, reslizumab) and IL-5R (benralizumab) have been reported to dramatically decrease blood eosinophil counts in asthma patients [58–60] and in patients with hypereosinophilic syndrome (HES) [61]. HES patients with skin involvement usually present with severe pruritus. Treatment of HES patients with mepolizumab and reslizumab has been shown to lead to a reduction of itch intensity along with decreased eosinophil counts [62, 63]. Similar effects were observed in a patient with Wells syndrome, another eosinophilic skin disease [64]. (Table 1).

Currently, there is only little data available for a potential role of eosinophil-mediated pruritus in CTCL. Nevertheless, eosinophil infiltration was detected in the skin of MF patients who presented with pruritus, but not in those without pruritus [65]. Furthermore, the group of patients with intense pruritus exhibited a significantly higher number of eosinophils that infiltrated the MF skin [65]. In addition, a positive correlation was observed between the presence of eosinophils in MF lesions and the disease stage [66]. Eosinophil presence is rare in the early stages of MF, but becomes a common characteristic in advanced stages [66]. The efficacy of biologics targeting IL-5 or IL-5R has not yet been explored in the treatment of CTCL-associated pruritus.

### Interleukin-25

Interleukin-25, also known as IL-17E, belongs to the family of IL-17 cytokines along with IL-17A-F [67, 68]. It is produced by activated Th2 cells, eosinophils, basophils, mast cells, and macrophages [69]. IL-25 signals through a heterodimer complex consisting of IL-17 receptor A (IL-17RA) and IL-17 receptor B (IL-17RB) [70, 71]. The IL-17RB mRNA expression seen in naïve T cells, Th2 and Th9 cells indicates that these cells may be IL-25 targets [70, 72]. In addition, skin macrophages, in particular of the M2 phenotype, and keratinocytes are also targets of IL-25 [73]. (Fig. 2).

IL-25 has been suggested to be involved in pruritus in AD by mutual upregulation with endothelin-1 [74], a potent pruritogen in human and mice [75–78]. In line with this, plasma endothelin-1 and serum IL-25 levels have been found to strongly positively correlate with itch intensity in AD and to be significantly elevated as compared to healthy control subjects [79, 80].

There is not much known about the connection of IL-25 and itch in CTCL. In patients with advanced disease, expression of IL-25 in keratinocytes and serum levels of IL-25 were significantly higher than in healthy control subjects [81], which also correlated with serum lactic acid dehydrogenase levels, a disease severity marker of MF and SS [81, 82]. However, the relationship between IL-25 levels in the lesions or serum of CTCL patients and the severity of pruritus is, as of yet, unknown.

### Interleukin-31

Interleukin-31 is a member of the IL-6 cytokine family and is thought to be mainly produced by activated Th2 cells, but also by other cells such as mast cells, macrophages, and dendritic cells [83, 84]. IL-31 signals via a heterodimeric receptor complex, which is composed of IL-31RA and the oncostatin M receptor β (OSMRβ) [85]. The IL-31R complex is expressed by many cell types, including T cells, keratinocytes, dendritic cells, eosinophils, macrophages, and dorsal root ganglia [86]. (Fig. 2).

IL-31 is thought to be importantly involved in the pathophysiology of chronic pruritus associated with various dermatological diseases. For example, in both stasis dermatitis and scabies, increased numbers of IL-31-producing M2 macrophages in the lesion have been linked to the severe pruritus in these patients [87, 88]. Furthermore, in patients with allergic contact dermatitis, serum levels of IL-31 are significantly higher as compared to healthy controls and correlate with the severity of pruritus [89]. A monoclonal antibody targeting the IL-31RA, nemolizumab, has been studied in AD and prurigo nodularis and was very effective in reducing pruritus in these patients [90–94]. (Table 1).

The information on the pruritogenic role of IL-31 in CTCL is conflicting. Some studies found serum levels of IL-31 to be significantly elevated compared to healthy controls [95–97], whereas another study showed that translational and transcriptional expression levels of IL-31 were very low or undetectable in CTCL patients [98]. One of the studies reporting increased serum IL-31 in CTCL did not observe a correlation with itch intensity [96], whereas the other two did [97, 99]. For example, Abreu et al. reported that, in CTCL patients with itch, IL-31 levels are higher than in those without and that the highest levels of IL-31 are found in those patients with severe itch (visual analogue scale of 6 or higher) [97]. Also, the level of IL-31 mRNA in peripheral blood mononuclear cells of CTCL patients have been found to be significantly increased and to correlate with the intensity of itch [99]. Additionally, the expression levels of IL-31, IL-31RA and OSMRβ in skin lesions of CTCL patients have been found to be increased, and the expression levels of IL-31 correlate with pruritus intensity [100]. The efficacy of nemolizumab has not yet been explored in the treatment of CTCL pruritus.

### CCL-1 and CCL-26

Chemokine CC motif ligand (CCL)-1 (also known as thymus-derived chemotactic agent 3) is a small glycoprotein and a typical chemokine, belonging to CC-type chemokines. CCL-26 (eotaxin-3) belongs to the eotaxin family, a CC chemokine subfamily that also includes CCL-11 (eotaxin-1) and CCL-24 (eotaxin-2) [101, 102]. CCL-1 is secreted by monocytes, activated macrophages and T lymphocytes. It is also expressed by dermal microvessels and epidermal antigen-presenting cells [103, 104]. CCL-26 is mainly produced by resident skin cells, including fibroblasts and smooth muscle cells, and is generally expressed only in non-hematopoietic cells [105]. CC chemokine receptor (CCR) 8 is the specific receptor of CCL-1, which most T cells in normal human skin express. It is also expressed by nerve cells and glial cells [103, 106]. CCR3, the receptor of CCL-26 [107], is highly expressed by eosinophils, with noted expression in basophils, Th2 cells, mast cells, and airway epithelial cells [108]. (Fig. 2).

Compared with healthy controls, serum CCL-1 and CCL-26 levels were significantly higher in patients with AD and bullous pemphigoid, both pruritic diseases [109–111].

In CTCL patients, serum CCL-1 and CCL-26 levels were significantly increased, especially in advanced cases [111, 112]. There is, furthermore, a significant correlation between serum levels of CCL1 and CCL26 with itch intensity in CTCL patients [113]. In addition, the expression of CCL26 mRNA in fibroblasts from skin lesions of CTCL patients is higher than in normal skin [112]. Currently, there are no therapeutic options available for testing the effects of CCL-1 and -26 targeted treatment of pruritus in CTCL.

### Thymic stromal lymphopoietin

Thymic stromal lymphopoietin (TSLP) is a member of the IL-2 cytokine family [114] and is mainly expressed by cells forming barrier surfaces, i.e. epithelial cells and keratinocytes [115]. The TSLP receptor is a heterodimeric receptor consisting of an IL-7 receptor α-chain and a common receptor-γ chain [116]. TSLP receptor mRNA has been found on many immune cell types, including dendritic cells, T cells, B cells, mast cells, natural killer T cells, and monocytes [117] (Fig. 2).

TSLP is held to be involved in the pathogenesis of pruritus in various dermatological diseases. For example, in dermatitis herpetiformis, skin-derived TSLP was shown to correlate with the intensity of pruritus [118]. A human monoclonal antibody specific for TSLP, tezepelumab, demonstrated significant but minor improvement in pruritus in moderate to severe AD patients as compared to placebo [119] (Table 1).

Compared with healthy controls, the expression level of TSLP in serum and lesions were significantly increased in CTCL patients, especially in the early-stage of the disease [120, 121]. However, the relationship between TSLP expression levels and CTCL pruritus are, as of yet, unclear and need further exploration. The efficacy of tezepelumab has not yet been explored in the treatment of CTCL pruritus.

## Neuropeptides and neurotrophins

### Nerve growth factor

**Fig. 3:**
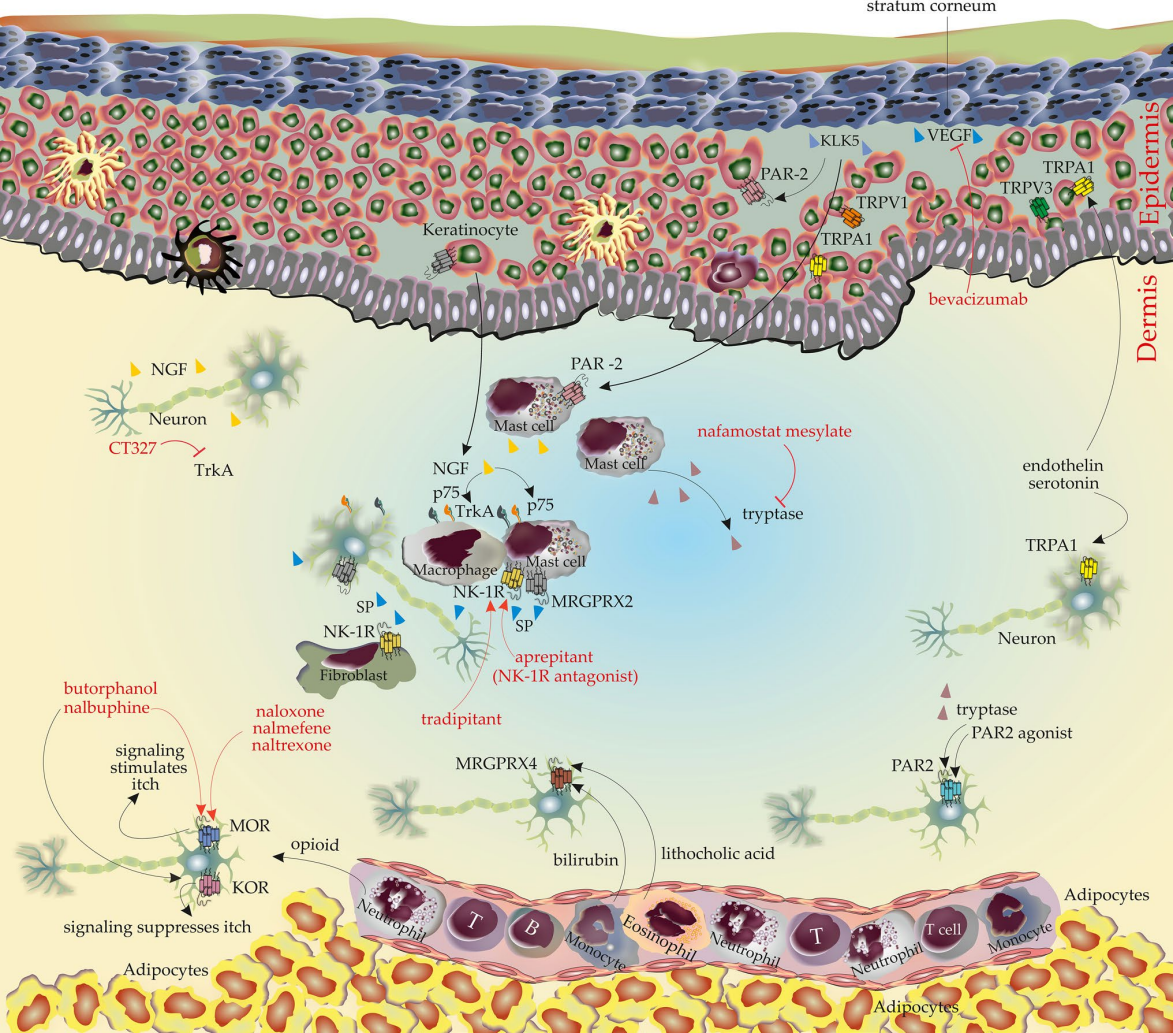
Neuropeptides, growth factors, and other substances and receptors potentially involved in CTCL pruritus. mAbs as treatment are shown in red with blocking symbol. All cells releasing the cytokines or express the receptors are shown for each subsection. Abbreviations: KLK5, Kallikrein Related Peptidase 5; KOR, k-type opioid receptor; MOR, µ -type opioid receptor; MRGPRX4, Mas-related G-protein coupled receptor member X4; NGF, Nerve growth factor; NK-1R, Neurokinin-1 receptor; PAR, Protease-activated receptor; SP, Substance P; TrkA, Tropomyosin receptor kinase A; TRP channels, Transient receptor potential channels; VEGF, Vascular endothelial growth factor

Nerve growth factor (NGF), together with brain-derived neurotrophic factor, neurotrophin-3 and neurotrophin-4/5, belongs to the family of neurotrophins [122, 123]. The production and maturation of NGF are accredited to a variety of cell types, such as keratinocytes, neurons and mast cells [124]. NGF binds to tropomyosin receptor kinase A (TrkA) with high affinity and to the p75 neurotrophin receptor (p75NTR) with low affinity [125]. In addition to nerve cells, many immune cells such as macrophages and mast cells also express NGF receptors and respond to NGF stimulation to induce a variety of effects that can be pro- or anti-inflammatory [126] (Fig. 3).

In patients with pruritic skin diseases including AD, prurigo nodularis and psoriasis, the levels of NGF in the plasma and expression of its receptors TrkA and p75NTR in lesional skin were significantly higher and associated with strong pruritus [127–129]. In line with this, significantly higher expression levels of NGF and TrkA were found in psoriasis patients with pruritus as compared to patients without pruritus, and the expression levels of NGF and TrkA were associated with pruritus severity [130, 131]. Furthermore, treatment of patients with psoriasis with the topical TrkA inhibitor CT327 was associated with a significant reduction of pruritus [132] (Table 1).

As for CTCL, patients with SS were reported to exhibit higher serum NGF levels as compared to healthy controls [113]. In addition, NGF-positive dermal nerve fibers were increased in the skin of these patients, while they were rarely detected in MF patients as well as healthy controls [113]. The efficacy of topical CT327 or of other NGF-targeting therapies has not yet been explored in the treatment of CTCL pruritus.

### Substance P

Substance P (SP) is a highly conserved peptide neurotransmitter that belongs to the tachykinin family [133]. Although mainly expressed by neurons, it is also expressed by non-neuronal cell types, such as microglia and immune cells [134]. The specific receptor of SP is neurokinin-1 receptor (NK-1R), a G protein-coupled receptor (GPCR). NK-1R is expressed by a variety of cells, including neurons, smooth muscle cells, fibroblasts, mast cells, T cells, B cells, and NK cells [135]. In addition, SP strongly activates Mas-related G-protein coupled receptor member X2 (MRGPRX2), a member of the Mas-related gene family, which is expressed in sensory neurons, mast cells, and keratinocytes [136–138] (Fig. 3).

SP and its receptors are thought to be involved in various dermatological and non-dermatological pruritic conditions. For example, plasma concentrations of SP were found to be elevated in AD patients as compared to healthy controls, and to correlate with pruritus intensity in these patients [139]. In patients with psoriasis, SP levels have also been shown to be elevated, and the number of SP-positive nerve fibers in lesional skin correlated with the severity of pruritus in these patients [131, 139, 140]. Compared to healthy skin and non-lesional skin, the number of SP-positive nerve fibers and expression of NK-1R were also significantly increased in other itchy diseases, such as chronic prurigo and chronic pruritus associated with internal diseases, drug-induced pruritus, brachioradial pruritus, and chronic pruritus of unknown origin. Levels of SP are also significantly increased in the blood of patients with chronic spontaneous urticaria [140–144].

Aprepitant, an NK-1R antagonist, has been reported to be an effective anti-pruritic drug in many case reports and case-series. Aprepitants’ antipruritic effects have been shown in patients with chronic pruritus, prurigo nodularis, brachioradial pruritus, drug-induced pruritus, paraneoplastic pruritus, and pruritus associated with systemic diseases such as chronic kidney disease, hyperuricemia and iron deficiency [145–153]. In randomized controlled trials in patients with chronic prurigo, microbial eczema, AD, pruritus and eczema craquelé, aprepitant, however, failed to significantly improve pruritus [154, 155]. Another NK-1R antagonist, serlopitant, was tested for the treatment of pruritus associated with prurigo nodularis (phase 2 trial positive, phase 3 negative), pruritus associated with psoriasis (phase 2 trial positive), CPUO (phase 2 trial positive), and pruritus associated with epidermolysis bullosa (phase 2 trial negative) [156–160]. Other NK-1R antagonists in clinical investigations as antipruritic drugs are tradipitant, which has shown some antipruritic effects in patients with mild AD [161], and orvepitant [162, 163] (Table 1).

In CTCL, serum levels of SP expression are significantly increased in patients as compared to healthy controls, and positively correlate with disease severity in MF patients [164]. The correlation of itch intensity and SP levels has, as of yet, not been assessed. Nevertheless, the efficacy of the NK-1R antagonist aprepitant has been explored in the treatment of CTCL pruritus and has shown a significant antipruritic effect in many case reports and case series [165–170] (Table 1). In SS, the results of a small randomized, double-blind, placebo-controlled crossover study did not support the antipruritic efficacy of aprepitant [171]. The authors acknowledged, however, that this study had several limitations, with one notable limitation being the recruitment of only 5 patients [171]. Furthermore, they attributed the differences in clinical response compared to previous studies to changes in disease activity and external factors, such as ambient temperature and humidity, which have the potential to influence the scoring of pruritus using the visual analog scale in patients with SS [171].

### Vascular endothelial growth factor

Vascular endothelial growth factors (VEGFs), also known as vascular permeability factors, are a family of growth factors, which consists of seven members, VEGF-A, -B, -C, -D, -E, and -F, and PlGF [172]. There are three types of VEGF receptors: VEGFR-1, 2, and 3, and different VEGFs have different affinities to different receptors [173].

Especially for VEGF-A, several observations support a role in pruritus in different conditions. For example, in psoriasis patients, expression of VEGF-A in lesional skin of patients with severe pruritus was higher than in those without pruritus [174]. In patients with AD, expression of VEGF-A in the epidermal stratum corneum was increased, and levels of VEGF were significantly higher in the serum and correlated with pruritus [175–177]. In chronic prurigo, VEGF-A immunoreactivity was markedly increased in the epidermis, dermis, and subcutis, which was associated with a marked increase in the number of blood vessels and epidermal thickness of prurigo lesions [178].

Bevacizumab, a VEGF-A inhibitor, was found to be effective in a patient with chronic prurigo, where it reduced pruritus [179] (Table 1). In addition, axitinib, an inhibitor of VEGFR-1-3, inhibits the scratching behavior seen in imiquimod-induced psoriasis mouse models [174].

In erythrodermic MF and SS, serum VEGF-A levels were significantly higher than those in healthy controls, and the levels significantly decreased after treatment, including topical and oral corticosteroids, ultraviolet phototherapy, oral etretinate, oral vorinostat and/or systemic chemotherapy. Furthermore, serum VEGF-A levels were significantly associated with the severity of pruritus in MF/SS patients [180]. However, the efficacy of bevacizumab has not yet been explored in the treatment of CTCL pruritus.

## Proteases

### Kallikreins

Kallikreins (KLKs) are a group of secreted serine proteases [181, 182]. In the skin, KLKs are mainly expressed in the upper stratum granulosum and stratum corneum [183, 184] (Fig. 3). There are at least 11 KLKs expressed in the epidermis, of which KLK5 is most abundant in the skin and may play an important role in itch [185, 186]. KLK5 can activate protease-activated receptor (PAR)-2, a GPCR expressed in a variety of skin cells, including sensory nerves, keratinocytes, and mast cells, which are thought to be involved in the elicitation of pruritus [187–189].

KLK5 activity was found to be increased in the skin of AD patients, and protein expression levels were significantly higher than those in healthy controls [190, 191]. In an animal experiment, mice injected with KLK5 exhibited significantly increased scratching behavior relative to vehicle controls [192].

KLK5 may also be involved in MF-associated pruritus. A study with 37 MF patients showed that the protein expression levels of KLK5 increased with the severity of pruritus [65].

### Tryptase

Tryptase is one of the major serine-proteinases and is secreted mainly by tissue mast cells and, to a lesser extent, also basophils [193, 194] (Fig. 3). Two main types of mast cell tryptase have been described, α- and β-tryptase. While α-tryptase is constitutively released by mast cells as an inactive pro-enzyme, β-tryptase is stored in mast cell granules and is released upon their activation [195]. β-tryptase cleaves several extracellular substrates including extracellular matrix proteins, activates PARs, in particular PAR-2, and it is a useful serum marker for mast cell activation in anaphylaxis and anaphylactoid reactions [195, 196].

Tryptase is thought to be involved in pruritus associated with various diseases. For example, serum tryptase levels were increased in renal disease with pruritus, and the intensity of pruritus correlated significantly with tryptase levels [197]. Tryptase level were also increased in AD patients with moderate to severe pruritus [198]. The connection of tryptase and pruritus is further supported by the correlation of blood tryptase reduction in AD patients treated with fexofenadine, an antihistamine, with pruritus improvement [199] (Fig. 3) Enhanced levels of tryptase release and tryptase activity are related to itch in chronic dermatitis, P-phenylenediamine-induced itch, and ovalbumin allergy-induced itch in mice [200–202]. Nafamostat mesilate, an oral serin protease inhibitor, inhibits itch-associated responses in mice mainly through the inhibition of mast cell tryptase [203].

However, although there is strong evidence in support of a direct connection between tryptase and itch from various diseases, there is currently only one study that involved a small group of patients with MF. This study observed numerically higher serum levels of tryptase in MF patients with pruritus compared to those without pruritus [204]. Furthermore, since tryptase is a marker of mast cell activation, any association between pruritus and tryptase may reflect the role of mast cell activation and the consecutive release of other pruritus associated mediators. Studies on the role of tryptase and mast cells are needed and should be performed.

## Itch associated receptors and ion channels

### Mas-related G protein coupled receptors

MRGPRs, including MRGPRA to -H and MRGPRX, comprise a large family of seven transmembrane-domain receptors mainly expressed in sensory neurons of the dorsal root and, importantly, on mast cells [205–207]. Of these, the MRGPRX receptors (MRGPRX1-4) are primarily expressed in humans and held to induce pruritus [208] (Fig. 3).

MRGPRs can be activated by a large variety of substances and mediators, including numerous synthetic drugs and a number of neuropeptides. For example, chloroquine, a widely used anti-malarial drug, activates MRGPRX1 and induces itch [209]. IPDef1, a tick salivary peptide, can evoke itch by activating MRGPRX1 on dorsal root ganglion neurons, and the concentration of PAMP1-20, an MRGPRX2 agonists, was found to be elevated in the skin in allergic contact dermatitis [210]. Levels of MRGPRX2 mRNA were increased in pruritic skin of patients with AD and psoriasis [141]. MRGPRX4 is thought to be implicated in the transmission of cholestatic itch where bilirubin excites peripheral sensory neurons and elicits pruritus through binding to and activation of MRGPRX4 [211]. Transgenic mice expressing human MRGPRX4 scratched more upon injection of bile acids, which are increased in the blood of cholestatic patients [205].

The role and relevance of MRGPRs in CTCL pruritus is, as of yet, entirely unclear and needs to be investigated.

### Opioids

The endogenous opioid system is one of the human innate pain-relief systems and uses specialized opioid receptors [212]: µ-type opioid receptors (MOR) for endorphins, k-type opioid receptors (KOR) for dynorphins, and δ–type opioid receptors for enkephalins [213]. Interestingly, opioid receptors have been found to differently connect with itch. For example, KOR signaling suppresses itch, whereas MOR signaling can stimulate itch [214, 215] (Fig. 3). These findings are derived from experiments with selective agonists and antagonists for the individual receptors. For instance, MOR antagonists, such as naltrexone, nalmefene, and naloxone, can significantly relieve severe itching caused by several different diseases, including AD, uremia, psoriasis, chronic prurigo, cholestatic itch and lichen simplex chronicus [216–224]. Intrathecal injection of the MOR agonists morphine or DAMGO elicited dose-dependent scratching and pruritus in mice and humans [225–227]. KOR agonists, such as difelikefalin and nalfurafine, can markedly improve pruritus in chronic kidney disease patients undergoing dialysis and pruritus in chronic liver disease [228–236]. Nalbuphine, a KOR agonist and MOR antagonist, can prevent intrathecal morphine-induced pruritus and be effective against pruritus in prurigo nodularis and uremia [237–241]. Butorphanol, another KOR agonist and MOR antagonist, has been reported to reduce chronic pruritus associated with various dermatological, internal, and neurological diseases [242–247] (Table 1).

In almost all lymph nodes of patients with SS, in contrast to all other lymphoma patients, MORs were found to be highly expressed [36]. Naltrexone, an orally semisynthetic MOR antagonist, was demonstrated to be effective in suppressing pruritus in patients with CTCL [216, 248–250], and another MOR antagonist, naloxone, improved pruritus in a patient with MF [251]. In the same patient, however, exacerbation of pruritus occurred after treatment with naltrexone, which may reflect the complexity of the opioid system [251].

### Protease-activated receptor-2

PAR-2, a G-protein coupled receptor (GPCR), is activated by serine proteases such as trypsin and tryptase [252, 253]. It belongs to the group of PARs which also includes PAR-1, -3, and -4 [254]. PAR-2 is expressed by epithelial, endothelial, and smooth muscle cells, as well as by cells of the immune and nervous systems [255, 256] (Fig. 3).

PAR-2 is involved in the pathophysiology of many inflammatory diseases, including AD. In the skin of patients with AD, the number of PAR-2 positive nerve fibers is significantly increased, and intracutaneous injection of endogenous PAR-2 agonists causes enhanced and prolonged itch [198]. Interestingly, skin of patients with AD has been found to be presensitized for protease-induced itch [257]. In mice, PAR-2 agonists can also induce scratching behavior [200, 258]. Mice with epidermal overexpression of PAR-2 develop an enhanced spontaneous scratching [259, 260], whereas inhibition of PAR-2 activation by PAR2 inhibitors such as SAM-11 and PZ-252 suppresses scratching behaviour [200, 202, 261].

Immunohistochemistry demonstrated that the expression of PAR-2 in the skin of MF patients is higher than in healthy controls. However, there was no difference of PAR-2 expression in MF patients with different degrees of pruritus [65].

### Transient receptor potential channels

Transient receptor potential channels (TRP channels) are non-selective calcium-permeable cation channels that compose the TRP ion channel superfamily located on the cell membrane [262–265]. TRP channels are divided into seven subgroups based on protein homology: TRPC, TRPV, TRPM, TRPA, TRPN, TRPP, and TRPML. Among them, five have been proposed to play a role in itch: TRPA1, TRPV1, TRPV3, TRPV4, and TRPM8 [266, 267]. They are expressed in different cell types in the skin and nervous system, such as keratinocytes and dorsal root ganglion neurons [268] (Fig. 3).

TRPA1 is considered to be an important mediator for itch signaling in mice and humans [262, 264, 269–272]. Burn patients with pruritus had increased TRPA1 mRNA compared to burn patients without pruritus, and TRPA1 mRNA expression showed a positive correlation with the intensity of post-burn pruritus [273]. Overexpression of TRPV 1 in pruritic skin was found to be positively correlated with itch intensity ratings in both AD and psoriasis patients [141]. Numerous clinical trials have confirmed that topical application of capsaicin – a TRPV1 agonist – is effective in reducing chronic pruritus of unknown origin [274, 275], and PAC-14028, a TRPV1 antagonist, showed a trend towards improvement of pruritus in AD patients in a phase 2b clinical trial [276, 277] (Table 1).

TRPV3 is implicated in itch in many skin diseases, including Olmsted syndrome and AD [278–280]. TRPV3 mRNA expression is higher in AD patients with pruritus than AD patients without pruritus and healthy controls [281, 282]. In burn patients, TPRV3 was significantly elevated in the epidermis of burn scars with pruritus when compared with burn scars without pruritus and was positively correlated with the intensity of pruritus [273]. A TRPV3 activator, carvacrol, has been reported to cause pruritus in humans [283, 284].

TRPV4 is also involved in a variety of pruritic conditions [285–287]. Like TRPV3, TRPV4 mRNA expression was increased in burn patients with pruritus compared to burn patients without pruritus and normal skin, and is positively correlated with the intensity of pruritus [273]. In numerous mouse disease models (psoriasis, allergic contact dermatitis and dry skin) and models using pruritus-inducing substances and TRPV4 agonists, a role for TRPV4 in itch induction has been confirmed [268, 285, 288–293].

Activation of TRPM8 induces a long-lasting cooling effect in the skin, and the application of cold is a well-known remedy for pruritus in many conditions [294]. TRPM8 agonists such as cryosim-1, menthoxypropanediol, and icilin can significantly improve recalcitrant pruritus associated with many diseases, including eczema, urticaria, AD, lichen sclerosus et atrophicus, and scalp itch [295–299].

TRP channels may also be involved in CTCL-asociated pruritus. The use of a CTCL mouse model demonstrated that one of the itch mediators in CTCL, miR-711, induced itching through direct activation of TRPA1 on sensory neurons, and this pruritus was decreased in TRPA1-knockout mice [300]. The efficacy of PAC-14028 or other TRP antagonists has not yet been explored in the treatment of CTCL pruritus.

## Conclusion

Chronic pruritus is complex, involves different pathways, and is likely to be different between diseases [301–303]. Although remarkable progress is being made in exploring the pathogenesis of pruritus, the underlying pathophysiology in CTCL-associated itch remains largely elusive. Here, we summarized and discussed the published evidence for a variety of mediators and receptors held be involved in itch associated with CTCL. In some instances, the evidence is rather circumstantial and requires investigations in CTCL patients or mouse models. In others, a relevant role in CTCL is supported by correlations of itch intensity and mediator levels. Although it is too early to say which mediators and receptors that drive pruritus in CTCL, the significant involvement of proteases (primarily tryptase), and neuropeptides (mainly SP) in the development and severity of pruritus in various dermatological diseases, including CTCL, suggests their potential as key players in this context. At the receptor level, cytokine receptors, MRGPRs and TRP channels are most likely important, and future drug development should target these receptors for the treatment of CTCL pruritus.

Currently, CTCL-associated itch is difficult to treat and has substantial impact on quality of life in these patients [8, 10, 304, 305]. Therefore, novel, effective and safe treatment options for pruritus in CTCL are desperately needed. The publication of further case reports and series is encouraged, but what we really need are controlled clinical trials.

## Data Availability

Data sharing not applicable to this article as no datasets were generated or analysed during the current Review.

## Abbreviations

AD: Atopic dermatitis
CBCL: Cutaneous B-cell lymphomas
CCL: Chemokine C–C motif ligand
CCR: CC chemokine receptor
CTCL: Cutaneous T-cell lymphomas
GPCR: G protein-coupled receptor
HES: Hypereosinophilic syndrome
IL: Interleukin
KLKs: Kallikrein-related peptidas
KOR: K-type opioid receptor
MF: Mycosis fungoides
MOR: µ -Type opioid receptor
MRGPRs: Mas-related G protein-coupled receptors
MRGPRX2: Mas-related G-protein coupled receptor member X2
NGF: Nerve growth factor
NK-1R: Neurokinin-1 receptor
OSMRβ: Oncostatin M receptor β
PAR: Protease-activated receptor
SS: Sézary syndrome
SP: Substance P
TrkA: Tropomyosin receptor kinase A
TRP channels: Transient receptor potential channels
TSLP: Thymic stromal lymphopoietin
VEGF: Vascular endothelial growth factor
